# Simultaneous Organic and Inorganic Host‐Guest Chemistry within Pillararene‐Protein Cage Frameworks

**DOI:** 10.1002/chem.202104341

**Published:** 2022-02-02

**Authors:** Ahmed Shaukat, Eduardo Anaya‐Plaza, Ngong Kodiah Beyeh, Mauri A. Kostiainen

**Affiliations:** ^1^ Department of Bioproducts and Biosystems Aalto University 02150 Espoo Finland; ^2^ Department of Chemistry Oakland University 146 Library Drive Rochester MI 48309-4479 USA

**Keywords:** host-guest chemistry, protein cages, protein crystals, pillararene, water remediation

## Abstract

Supramolecular self‐assembly of biomolecules provides a powerful bottom‐up strategy to build functional nanostructures and materials. Among the different biomacromolecules, protein cages offer various advantages including uniform size, versatility, multi‐modularity, and high stability. Additionally, protein cage crystals present confined microenvironments with well‐defined dimensions. On the other hand, molecular hosts, such as cyclophanes, possess a defined cavity size and selective recognition of guest molecules. However, the successful combination of macrocycles and protein cages to achieve functional co‐crystals has remained limited. In this study, we demonstrate electrostatic binding between cationic pillar[5]arenes and (apo)ferritin cages that results in porous and crystalline frameworks. The electrostatically assembled crystals present a face‐centered cubic (FCC) lattice and have been characterized by means of small‐angle X‐ray scattering and cryo‐TEM. These hierarchical structures result in a multiadsorbent framework capable of hosting both organic and inorganic pollutants, such as dyes and toxic metals, with potential application in water‐remediation technologies.

## Graphical abstract

**Very accepting**: (Apo)ferritin protein cages self‐assemble with cationic pillararenes into porous crystalline framework structures. The apoferritin and pillararene in the crystals can simultaneously host inorganic and organic pollutants, where the binding is determined by the host‐guest chemistry.

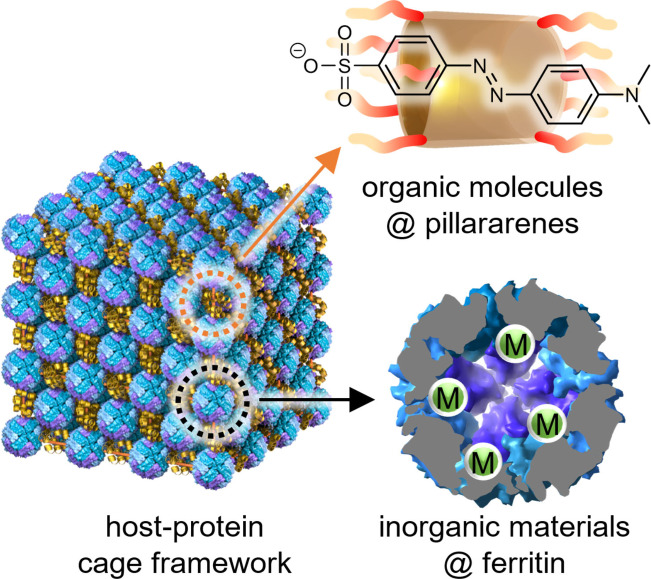

The design and development of hierarchical structures based on the bottom‐up assembly of biological building blocks has received growing interest in nanotechnology and material science. With the correct choice of building blocks, the resulting highly ordered (crystalline) structures can be engineered to present a porous structure with well‐defined pore sizes similar to metal–organic frameworks (MOFs),^[1]^ covalent organic framework (COFs),^[2]^ perovskites,^[3]^ and zeolites.^[4]^ Such porous structures hold the potential to be applied in technologies including drug delivery, water remediation, and catalysis.[^5^, ^6^, ^7^, ^8^, ^9^, ^10^] Among the plethora of biomolecules available, self‐assembled nanocompartments such as protein cages are of high interest due to their uniform size and shape.[^11^, ^12^, ^13^, ^14^, ^15^, ^16^] Together with their ability to encapsulate cargo, protein cages provide biocompatibility and biodegradability with chemical and thermal stability.[^17^, ^18^, ^19^] Protein cages have been extensively exploited as reaction vessels, templates for materials synthesis, drug carriers, bioimaging, and vaccine development.[^19^, ^20^, ^21^, ^22^, ^23^] Among many protein cages, ferritin (Ft) has emerged as an exceptional and promising building block. The iron oxide‐containing cage can be found in eukarya, archaea, and bacteria and plays a vital role in iron regulation. The iron‐free cage (apoferritin, aFt) presents an inner cavity, which can act as a host for a variety of organic and inorganic materials.^[24]^ Due to their uniform shape and size, both cages, that is, Ft and aFt have been ideal candidates to be organized into multifunctional higher‐order superlattices. They have already been used to create higher‐order crystalline structures by self‐assembly,^[25]^ together with organic dyes,^[26]^ dendrons and dendrimers,[^27^, ^28^, ^29^] polypeptides and proteins,[^25^, ^30^, ^31^] metal ions or nanoparticles,[^32^, ^33^, ^34^, ^35^] and macrocyclic molecules^[36]^ with varying potential applications in catalysis,[^31^, ^37^] storage, and separation. Even though proteins are known for their relatively low stability outside aqueous media, the structural robustness of these materials in dry state has been previously demonstrated.^[32]^ Their stability can also be increased by means of small molecule crosslinking,[^38^, ^39^] or embedding the structure within polymeric^[40]^ or inorganic matrixes.^[41]^

Macrocycles such as cyclophanes have a cavity of defined size that can bind guest molecules through noncovalent interactions with high selectivity and affinity. Pillar[*n*]arenes are composed of *“n*” hydroquinone units that adopt a barrel‐shaped configuration with variable cavity dimensions depending upon the number of hydroquinone units.[^42^, ^43^] A critical parameter for the host‐guest chemistry of pillararenes is the derivatization of the hydroquinone units. Versatile functionalization of pillar[*n*]arenes can provide various modified derivatives with anticipated chemical or physical properties for a wide range of applications in supramolecular chemistry.[^44^, ^45^, ^46^, ^47^, ^48^] There have been several advances in the controlled assembly of proteins with macrocyclic compounds such as calixarenes acting as molecular glue to yield crystalline frameworks.[^49^, ^50^, ^51^] Recently, we have studied the electrostatic self‐assembly of *Pyrococcus furiosus* aFt and a library of cyclophanes.^[36]^ An optimal cyclophane was identified, which formed crystalline porous framework through tuning the electrostatic interactions. However, insights into the practical implications of the structures with two differentiated chemical environments have not been described.

In this article, a protein crystal combining two types of molecular hosts is presented. On one hand, aFt and Ft present the ability to naturally host ionic metals. We studied abundantly available horse spleen Ft and aFt (Figure 1a), which are composed of a different ratio of the heavy chain (21 kDa) and light chain (19 kDa) subunits depending upon the type of tissue, and present a pI of ∼5.4. The cavity can accommodate up to ∼4500 Fe atoms in the form of a hydrous ferric oxide mineral core with a variable amount of phosphate.^[52]^ The size of the outer shell of ferritin is 12 nm, while the inner cavity is approximately 8 nm in size (Figure 1a). On the other hand, pillar[5]arene (**P10+**), a cyclophane with ten positive charges, was used as a molecular glue. **P10+** presents high affinity and selectivity to hydrophobic moieties due to the hydrophobic cavity with an inner diameter of approximately 1.5 nm (Figure 1b). Such porous materials have added advantage of improved mass diffusion properties and can be used to develop efficient catalysts and scavengers for water remediation purposes. We have characterized these cyclophane–protein cage frameworks (CPF) through different techniques, which detail the electrostatically driven self‐assembly into highly symmetrical order structures. The CPF adopts a crystal structure where protein cages form a face‐centered cubic (FCC) lattice, and the pillar[5]arene hosts are electrostatically bridging the protein lattice and provide additional functionality for the pores between the proteins. Furthermore, CPFs demonstrate selective host‐guest chemistry by binding common water pollutants such as methyl orange and heavy metal ions.

**Figure 1:**
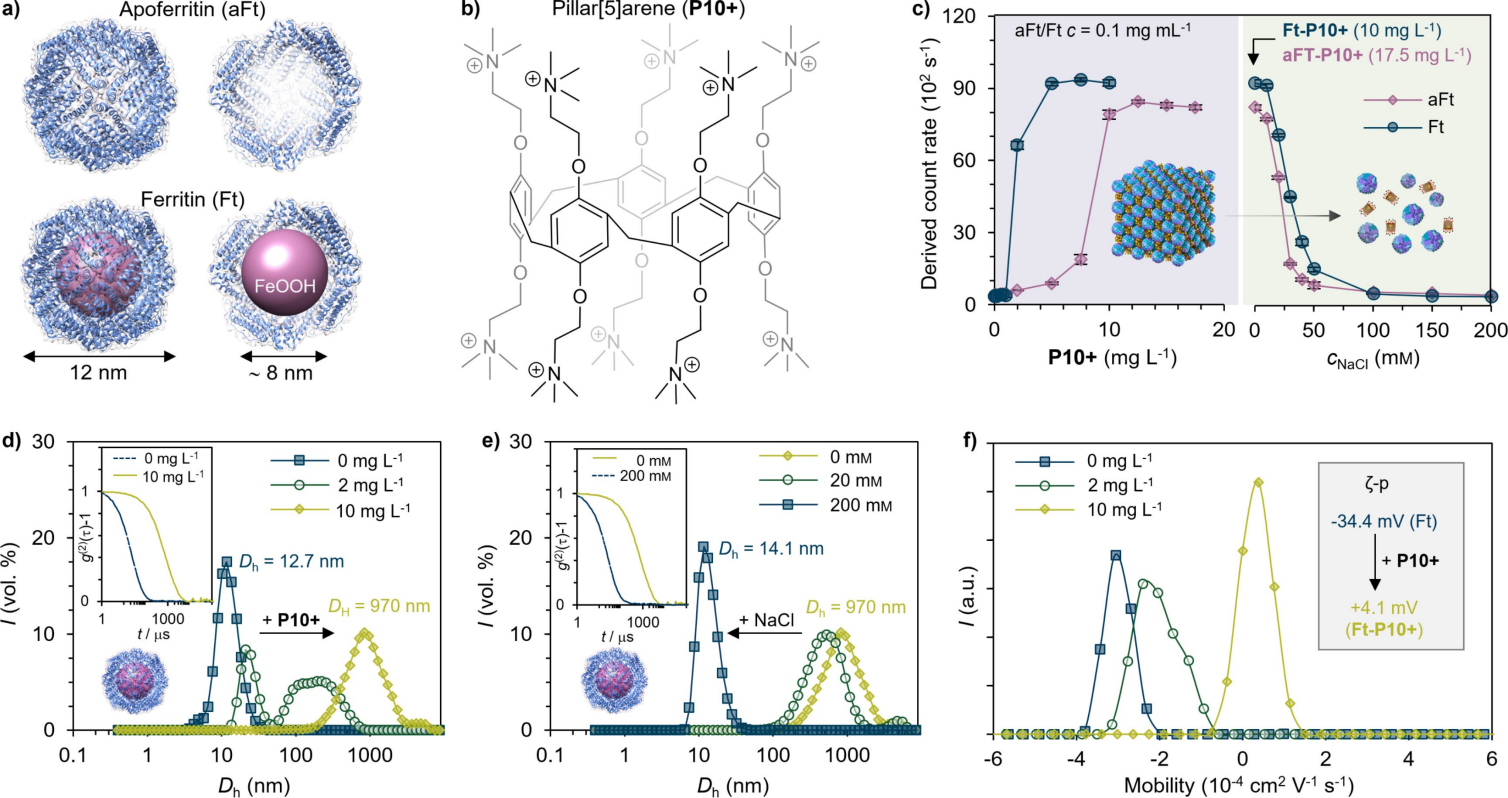
Self‐assembly of protein cage–**P10+** complexes. a) Structure of the horse spleen apoferritin (aFt; PDB ID: 2 W0O; top) and ferritin (Ft; bottom) cages viewed along the fourfold symmetry axis. The images on the right show the core of the protein cages; for aFt, the core is empty, whereas Ft has an iron core. b) Chemical structure of the synthetic host pillar[5]arene (**P10+**) used in this study. c) Left: Dynamic light scattering (DLS) of Ft and aFt solutions titrated with an increasing concentration of **P10+**; this shows the complexation of both protein cages. Right: The complexes are disassembled by increasing the ionic strength of the medium by addition of NaCl. d) DLS data for the volume‐averaged size distribution of free Ft titrated with an increasing amount of **P10+** and e) the resulting complexes disassembled with NaCl. Insets: second‐order autocorrelation functions of the corresponding measurements. f) Electrophoretic mobility and *ζ*‐p measured for **Ft**–**P10+** complexes.

The synthesis of **P10+** has been described elsewhere.^[36]^ The formation of large complexes between the protein cages (Ft and aFt) and the molecular host **P10+** was first studied using dynamic light scattering (DLS) by monitoring the increase in particle count rate as well as the hydrodynamic diameter (*D*_h_). A constant concentration of aFt or Ft (0.1 mg mL^−1^) in Tris buffer (20 mm, pH 7.5) was titrated with an increasing concentration of **P10+** (Figure 1c, left). First, Ft shows a sharp intensity increase, which corresponds to the presence of aggregates. This is achieved at [**P10+**] of 10 mg L^−1^, whereas for aFt, it is achieved at 17.5 mg L^−1^. The small variation in the point of aggregation can be explained by the significantly smaller molecular weight of aFt due to the absence of the iron oxide core, which results in a higher number of cages per volume. Therefore, a higher **P10+** concentration is required to induce complex formation. To prove the electrostatic interaction as the key driving force for recognition,[^53^, ^54^] the complexes are disassembled by increasing [NaCl] for both protein cages (Figure 1c, right). This process occurs at similar [NaCl] (i. e., above 50 mm) for both cages. Secondly, Ft shows a change in *D*_h_ (Figure 1d), which suggests that when the concentration of **P10+** increases, there is an increase in the hydrodynamic diameter. We observed the decrease of the peak at 12.7 nm, which corresponds to the native size of Ft. At the same time, new peaks emerge at higher **P10+** concentrations and reach approximately 1000 nm at [**P10+**]=10 mg L^−1^, which corresponds to **Ft–P10+** complexes. Similarly, with the addition of NaCl (Figure 1e), the hydrodynamic diameter reverts to a similar size to native Ft (14.1 nm) which shows that the system can be efficiently disassembled to the original state by simply increasing the electrolyte concentration. The electrophoretic mobility and *ζ*‐potential (*ζ*‐p) measurements presented in Figure 1f confirm the transition of an overall negative surface charge of free Ft particles (*ζ*‐p=−34.4 mV), to an overall positive charge when the complexes are formed. Similar results for *D*_h_, electrophoretic mobility and *ζ*‐p were observed with **aFt–P10+** complexes (Figure S1 in the Supporting Information).

Small‐angle X‐ray scattering (SAXS) was used to determine the morphology of aFt and Ft CPFs. All samples were formed by mixing a constant concentration of aFt or Ft (4 mg mL^−1^) with **P10+** (2.67 mg mL^−1^) in Tris buffer (20 mm, pH 7.5) with varying NaCl concentration. This results in cloudy solutions due to the presence of large assemblies, that were allowed to sediment for one hour in refrigerator. The measured SAXS patterns show clear Bragg reflections (Figure 2a, b). The **aFt–P10+** complex shows well‐resolved diffraction peaks at low NaCl concentrations (0–40 mm). However, increasing the electrolyte concentration results in a decrease in the attractive interactions and prevents assembly formation, which is also in good agreement with the DLS data. With **Ft–P10+** complexes (Figure 2b), the Bragg reflections were observed even at higher ionic strength (up to 90 mm). In both cases, the relative peak positions fit with the first allowed reflections of a FCC lattice (Figures 2c and S2). They are assigned to the (111), (200), (220), and (311) reflections, which correspond to *q_hkl_*/q*=√3, √4, √8, and √11, respectively. Plotting the square root of the sum of the squared Miller indexes (√(*h* ^2^+*k* ^2^+*l* ^2^)) versus the assigned magnitude of the scattering vector (*q*) results in a linear trend that yields a slope of 26.69 *q* (Å^−1^), and for the fitted FCC system, a lattice parameter of *a*_SAXS_=18.6 nm (Figure 2c, inset). The calculated nearest‐neighbor aFt center‐to‐center distance is 13.2 nm, which is in good agreement with the reported aFt diameter (*d*_aFt_ ∼12 nm) and the observed DLS dimensions (14.7 nm; DLS data in Figure S2). Also, the comparison to a simulated scattering curve from a finite FCC structure confirms the Bravais lattice space group *Fm* $\bar{3}$ *m* (no. 225). Similar lattice constant and dimensions were determined for **Ft–P10+** complexes (*a*_SAXS_=18.2 nm and calculated nearest‐neighbor Ft center‐to‐center=13.1 nm) and is presented in Figure S2.

**Figure 2:**
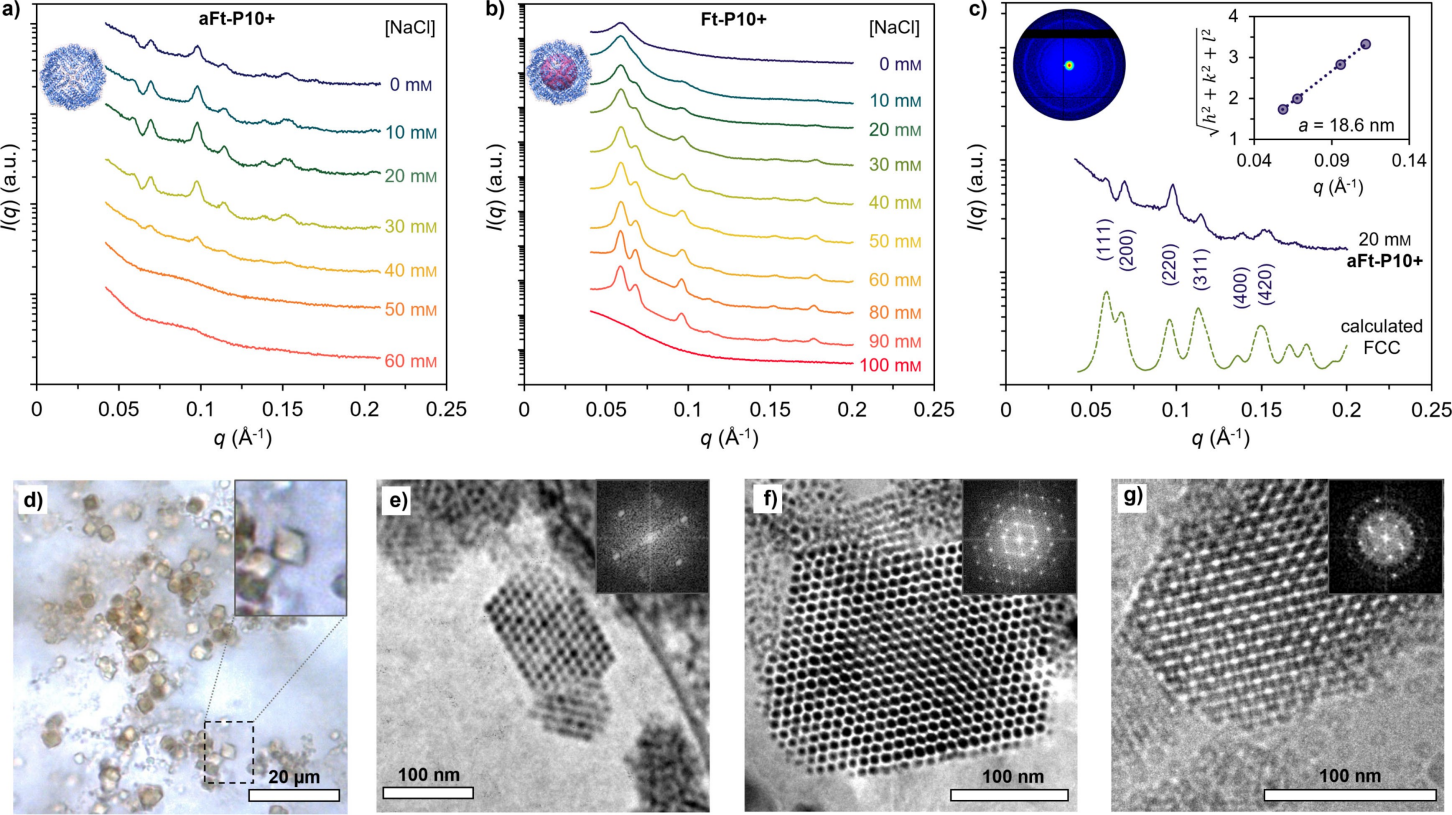
Structural characterization of **(a)Ft–P10+** complexes. SAXS diffractograms measured for a) **aFt–P10+** and b) **Ft–P10+** complexes at various NaCl concentrations. c) SAXS data for the **aFt–P10+** complex at 20 mm of NaCl, compared to the fitted FCC model (offset in the *y*‐direction for clarity). Inset: Square root of the sum of the square of the Miller indexes of the assigned reflections for the FCC structure vs. the measured *q*‐vector positions. Unit cell parameter of *a*=18.6 nm (space group *Fm* $\bar{3}$ *m*, no. 225). The concentrations of the protein cages (aFT and Ft) and **P10+** are constant in all SAXS experiments, that is, 4 and 2.67 mg mL^−1^, respectively; they were mixed in 20 mm Tris buffer (pH 7.5). d) Optical microscopy image of **Ft–P10+** crystals showing octahedral habit and sizes over 5 μm. Cryo‐TEM image of vitrified aqueous solutions containing e) **Ft–P10+** with 20 mm NaCl, f) **Ft–P10+** with 80 mm NaCl, and g) **aFt–P10+** with 20 mm NaCl. Insets: Corresponding fast Fourier transform (FFT).

By using the hanging drop method (Figure S3), **Ft–P10+** crystals with octahedral habit and dimensions of approximately 5 μm were observed (Figure 2d). For all other protein cage complexes at different NaCl concentrations, no faceted macrocrystals were observed under optical microscopy (Figure S4). To visualize and further analyze the formed lattices in solution, cryogenic transmission electron microscopy (cryo‐TEM) was used. A vitrified sample from the freshly prepared solution of both CPFs, that is, **Ft–P10+** and **aFt–P10+** was imaged (see the Supporting Information for sample preparation). After optimizing the crystallization conditions for microscopy, **Ft–P10+** complexes were imaged at 20 mm and 80 mm of NaCl based on better crystallinity observed through SAXS results. A similar selection was also chosen for **aFt–P10+** with 20 mm NaCl. Figure 2e–g shows typical small crystallites with a highly ordered arrangement of individual Ft or aFt particles. Low magnification images are shown in Figure S5. Note that samples were prepared at final protein concentration of 4 mg mL^−1^, and subsequently diluted to 1.33 mg mL^−1^ after crystal formation, indicating that the structures remain largely unchanged upon dilution.

The pore size of **Ft–P10+** crystals (*d*_oct_ = 6.2 nm; Figure S6) allows hosting of relatively large functional moieties within the interstitial space of the crystalline structure. In the CPFs, the voids between the protein cages are only partially occupied by the **P10+** bridging the proteins, thus being available for binding suitable small‐molecular guests (Figure 3a). The Ft cage, on the other hand, is well known for its ability to oxidize, nucleate, and host various metal ions,^[55]^ which has been utilized for water treatment solutions even at industrial scale.[^56^, ^57^] To test our hypothesis, the selective binding of organic guest molecules was initially studied by following the changes in the absorption spectra of two dye molecules: methyl orange (MO) and fluorescein. MO is also a typical dyeing agent in the fabric industry as well as a potentially carcinogenic pollutant, and therefore a relevant model molecule for water remediation applications.^[58]^ Further, the dimensions of MO are suitable for binding inside cationic pillararenes, displaying a solvatochromic effect, whereas fluorescein can fit the cavity only partially.

**Figure 3:**
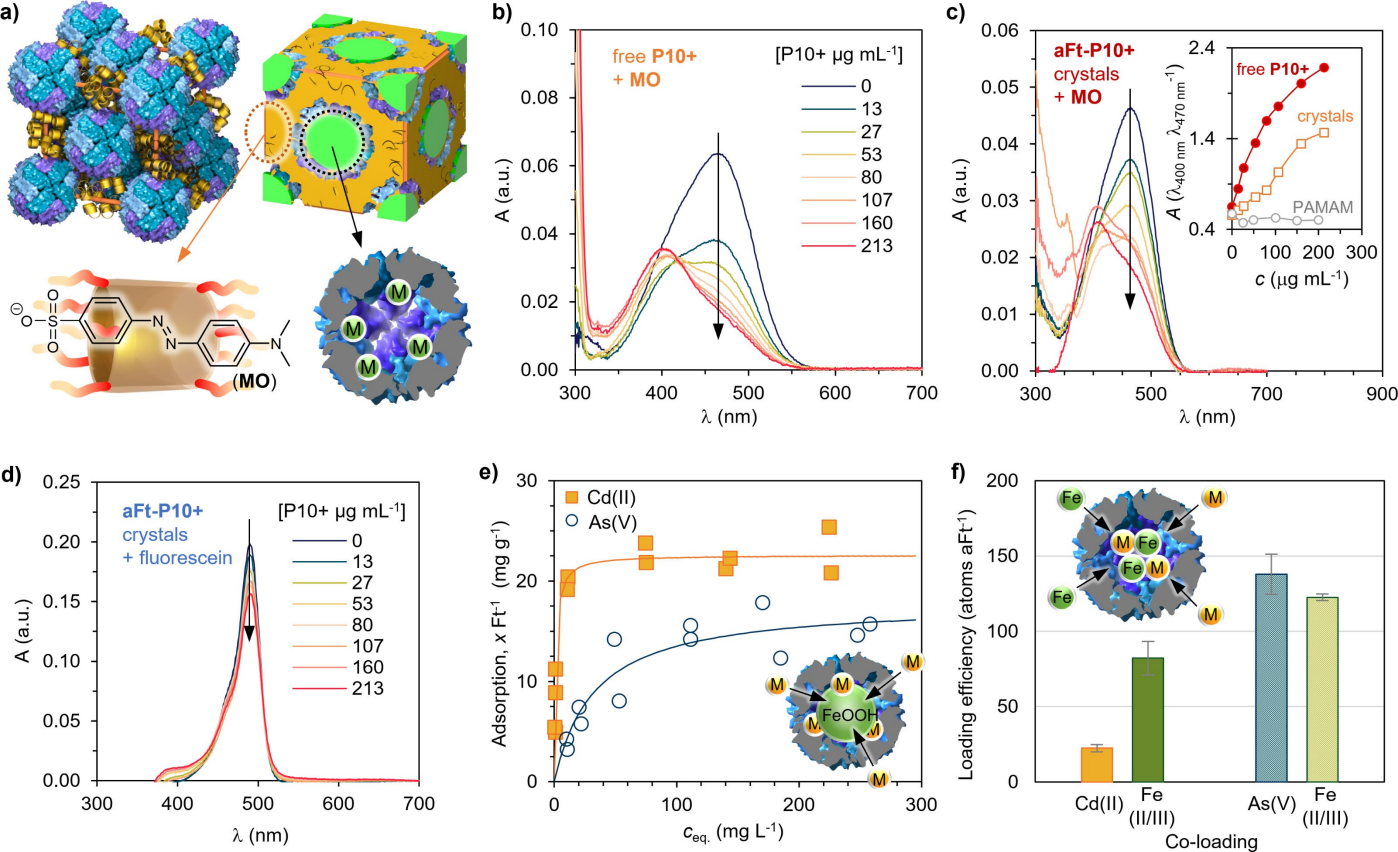
Host‐guest binding of organic and inorganic materials. a) Schematic presentation of the **Ft–P10+** composite unit cell showing full particles (top left) and highlighting the different areas for the simultaneous binding of small organic molecules by the cyclophane (yellow, bottom left) and inorganic materials inside the ferritin cage (green, bottom right). UV‐Vis titration of **MO** (2.5 μg mL^−1^) with b) **P10+** (*c*_**P10+**_=0–213 μg mL^−1^) and c) **aFt–P10+** (*c*_**P10+**_=0–213 μg mL^−1^) shows clear binding. Inset: 400/470 nm absorbance ratio for **P10+**, **aFt–P10+** crystals, and **aFt‐PAMAM‐G2** crystals. The last‐named, showing no binding, is plotted as a reference. d) Control titration of fluorescein (2.5 μg mL^−1^), which is too large to bind efficiently inside the **P10+** cavity (*c*_**P10+**_=0–213 μg mL^−1^), shows very minor changes in the spectrum. e) Adsorption isotherms of Cd^II^ and As^V^ on **Ft–P10+** complexes obtained by inductive coupled plasma–optical emission spectrometry (ICP‐OES). Solid lines present Langmuir adsorption isotherm fits. Inset: schematic presentation of the Cd^II^ and As^V^ (generalized as M) adsorption on the ferritin mineral core. f) Elemental analysis of aFt co‐loaded with Fe and Cd^II^ or As^V^. Inset: a schematic presentation of the co‐loading inside aFt.

When MO is titrated with free **P10+**, a clear decrease in the UV‐Vis absorption spectrum of MO is observed at 470 nm, together with an increase at 400 nm; this indicates that the MO is binding inside the host (Figure 3b). Such changes are in good agreement with previously reported observations of MO‐pillar[5]arene complexes.^[59]^ Binding inside the cavity is further supported by i) the broadening of the MO and **P10+** NMR signals caused by the interaction, ii) the shielding of the aromatic signals of MO up to +0.88 ppm (Figure S8a), and iii) the deshielding of the aromatic signals of the host upon intake.^[60]^ A similar change in absorption is observed when the titration is carried out by using the **aFt–P10+** crystals. Extracting the crystal‐induced background scattering (Figure S9b) yields similar changes in the absorption spectra (Figure 3c). Plotting the absorbance ratios against the host concentration indicates that the crystals are somewhat less efficient binders compared to freely soluble host in terms of the **P10+** concentration. This can be expected since the binding of aFt to **P10+** can negatively influence the host's ability to accept guests. However, binding inside the crystals still clearly takes place. If the aFt crystals are formed with a generation two polyamidoamine dendrimer (PAMAM−G2), which is not able to complex MO, there are no changes in UV‐Vis spectra during the titration (Figure S9i). The UV‐Vis titration data show only minor changes for the fluorescein absorbance upon binding to the **P10+** (Figure 3d). Together with the very limited shift of the NMR signals of fluorescein (Figure S8b), the results verify our hypothesis that small molecules can be selectively bound by the porous CPFs albeit with some loss of efficiency compared to free hosts, provided that the host is able to interact efficiently with the guest molecule.

To exploit the ferritin cavities in the complexes, we studied their ability to bind toxic metal ions such as arsenate. This is relevant as groundwater contaminated by cadmium and arsenic is a major problem, especially in South (East) Asia, and typical purification methods rely on the binding or co‐precipitation with iron oxide or hydroxides.^[61]^ Native ferritin hosts possess an amorphous iron hydroxide (FeOOH) core, which is known to adsorb heavy metals, such as arsenate and cadmium. This approach of binding arsenic was also used very recently for leukemia cells featuring high CD71 expression, which resulted in stronger cytotoxic effects than ionic arsenic alone.^[62]^ To study the loading of these inorganic materials, we turned to porous **Ft–P10+** complexes. Simultaneous binding of organic and inorganic guests was carried out by first loading the complexes with MO, and then batch sorption isotherms were measured for As^V^ and Cd^II^ by means of inductive coupled plasma—optical emission spectrometry (ICP‐OES). The samples were prepared by equilibrating a known concentration with a constant quantity of **Ft–P10+** crystals (Figure 3e). Fitting the measured adsorption data with Langmuir and Freundlich models showed a better correlation with the Langmuir model (Table S1) and yielded a maximum adsorption capacity of 24.5 and 17.8 mg g^−1^ for Cd^II^ and As^V^, respectively. Furthermore, it has been shown that iron Fe^II^ can be oxidized and deposited as Fe^III^ inside apoferritin simultaneously with different oxo‐anions.^[63]^ We were able to load the **aFt–P10+** CPFs with iron and Cd^II^ or As^V^ by using O_2_ as the oxidant. It was found that in the CPF state, each aFt cage takes in on average 82 Fe and 22 Cd atoms (Figure 3f). In contrast, when Fe is co‐loaded with As, the loading efficiency increases to 123 and 138 atoms per aFt, respectively. These unoptimized uptake amounts offer development potential as the theoretical maximum loading of iron atoms is approximately 4500 in eukaryotic ferritins. Considering further, genetic modification of ferritin would offer further routes to improve the metal uptake efficiency and specificity.

In conclusion, the molecular host **P10+** yields porous crystals with commercially available aFt and Ft by electrostatic self‐assembly. The interaction was successfully demonstrated by DLS, and the crystallinity of the complexes was further confirmed by means of SAXS, resulting in FCC‐packed crystalline frameworks with well‐defined porosity. The resulting crystals were thoroughly characterized by optical microscopy and cryo‐TEM. Host‐guest studies indicated that such composite crystals can bind efficiently organic (MO) and inorganic (Cd^II^ and As^V^) pollutants, enabled by the porous protein–molecular host network. We emphasize that these results not only demonstrate that the system is multifunctional but also pave the way for the successful design of protein assemblies with confined and synergetic chemical environments. Such supramolecular approaches and binding capabilities can be extended to other molecular guests like cyclodextrins, with a rich host‐guest chemistry.

## Conflict of interest

The authors declare no conflict of interest.

## Supporting information

As a service to our authors and readers, this journal provides supporting information supplied by the authors. Such materials are peer reviewed and may be re‐organized for online delivery, but are not copy‐edited or typeset. Technical support issues arising from supporting information (other than missing files) should be addressed to the authors.

Supporting Information

## Data Availability

The data that support the findings of this study are available from the corresponding author upon reasonable request.
