# Supplementary material for: Simultaneous Organic and Inorganic Host‐Guest Chemistry within Pillararene‐Protein Cage Frameworks
Source: Chemistry. 2022 Feb 2;28(11):e202104341. doi: 10.1002/chem.202104341 (PMC9305414; doi:10.1002/chem.202104341)
Supplement: Supplementary file 1 — Supporting Information [file CHEM-28-0-s001.pdf]

Chemistry–A European Journal

Supporting Information

Simultaneous Organic and Inorganic Host-Guest Chemistry within Pillararene-Protein Cage Frameworks

Ahmed Shaukat, Eduardo Anaya-Plaza, Ngong Kodiah Beyeh, and Mauri A. Kostiainen*

Table of contents

Chemicals S2
Methods S2
Dynamic light scattering (DLS) and ζ-potential measurements S2
Cryogenic transmission electron microscopy (cryo-TEM) S2
Small-angle X-ray scattering (SAXS) S2
UV-visible spectrophotometry (UV-vis) and host-guest titrations S3
Inductively coupled plasma optical emission spectrometry (ICP-OES) S3
Additional SAXS, DLS, and ζ-potential measurements S3
Additional Optical microscopy and TEM images S4
Optical microscopy S4
Guest binding studied by NMR Spectroscopy S6
Additional UV-Vis data S8
Isotherm parameters S9
Supplementary references: S9

Experimental section

Chemicals

All chemical reagents were purchased from Sigma-Aldrich unless otherwise stated and used without further purification. Tris base, PAMAM-G2, horse spleen derived apoferritin (aFt) and ferritin (Ft) were purchased from Sigma-Aldrich, fluorescein and methyl orange from Honeywell, and sodium chloride from VWR Chemicals. The water used in all the experiments was Milli-Q grade. The buffer utilized for all experiments was 20 mM Tris with 7.5 pH (adjusted with 1M HCl) unless otherwise stated.

Methods

Dynamic light scattering (DLS) and ζ-potential measurements

The hydrodynamic diameter (D_h) of the assemblies was measured using a Malvern Instruments DLS device (Zetasizer Nano ZS Series) with a 4 mW He-Ne ion laser at a wavelength of 633 nm and an avalanche photodiode detector at an angle of 173°. All experiments were carried at room temperature. PMMA cuvettes and dip cell probe (Malvern) used for the size and electrophoretic mobility measurements respectively. Zetasizer software (Malvern Instruments) was used to obtain the particle size distributions and ζ-potentials using the following sample preparation: 100 mg L^{-1} of protein cages (Ft and aFt) dissolved in buffer (20 mM Tris (pH 7.5)) was titrated with the **P10+** (0.1–17.5 mg L^{-1}) to reach the desired ratio (no dilution correction was done as the total addition did not exceed 5 % of sample volume), which was finally titrated with 0.01–0.5 M NaCl to disassemble the complex. For ζ-potentials measurements, milliQ water was used as the solvent.

Cryogenic transmission electron microscopy (cryo-TEM)

The cryo-TEM images were collected using JEM 3200FSC field emission microscope (JEOL) operated at 300 kV in bright field mode with an Omega-type zero-loss energy filter. The images were acquired with Gatan Digital Micrograph software while the specimen temperature was maintained at -187 °C. The cryo-TEM samples were prepared by placing 3 μL aqueous dispersion of the sample on a 200-mesh Lacey carbon film on Copper TEM Grids (agar scientific) and plunge-frozen into liquid ethane using Leica grid plunger with 3 s blotting time under 100 % humidity. The grids with vitrified sample solution were maintained at liquid nitrogen temperature and then cryo-transferred to the microscope. The TEM grids were plasma cleaned before use (NanoClean 1070, Fischione Instruments). Images were further processed using ImageJ software.

Small-angle X-ray scattering (SAXS)

The SAXS samples were measured using the Xenocs Xeuss 3.0 C device equipping with a GeniX 3D Cu microfocus source (wavelength λ = 1.542 Å) and EIGER2 R 1M hybrid pixel detector at a sample-to-detector distance of 0.6 m. One-dimensional SAXS data was obtained by azimuthally averaging the 2D scattering data and the magnitude of the scattering vector q is given by $q = 4\pi \sin\theta / \lambda$, where 2θ is the scattering angle.

Sample preparation for SAXS, cryo-TEM and UV-Vis

Cyclophane-protein cages framework (CPF) samples were prepared by combining 6 μL of aqueous protein cage solution (10 mg mL^{-1}), 3.5 μL 20 mM Tris pH 7.5 buffer, 1.5 μL of 0–1000 mM NaCl solution and 4 μL of 10 mg mL^{-1} cyclophane in water, in this order. The samples were gently mixed with a pipette. Immediately after mixing precipitates are formed. The samples are then incubated in refrigerator for 1 hour to settle down the precipitate and herafter the sediments is further analysed.

For Cryo-EM, in order to avoid overcrowding of sample on the TEM grid, the samples prepared from above mentioned procedure were further diluted three times in 20 mM Tris pH 7.5 buffer before preparation i.e., 1.33 mg mL^{-1} of (a)Ft .

UV-visible spectrophotometry (UV-vis) and host-guest titrations

The CPF samples were prepared as described above. The aFT-PAMAM-G2 crystals were prepared as in our previous publication.[1] The crystals were incubated in the refrigerator for 24 h for sedimenting the crystals. After the incubation, the supernatant was replaced with fresh buffer. For measurements, 100 µL of 20 mM Tris pH 7.5 buffer containing 2.5 µg mL^{-1} of the dye (methyl orange or fluorescein) was titrated with **aFt-P10+** crystals. The UV-Vis spectra were measured using a Cytation 3 plate reader (BioTek).

Inductively coupled plasma optical emission spectrometry (ICP-OES)

The metal content of the samples was analysed with inductively coupled plasma optical emission spectrometry using a Perkin Elmer ICP-AES Optima 7100 DV instrument.
For the isotherm measurements, **Ft-P10+** crystal solutions (containing: 1. 4 mM Tris pH 8, 20 mM NaCl; 2. 6.67 mg mL^{-1} Ft; 3. 2.67 mg mL^{-1} P10+ and 4. 2.3 µg mL^{-1} methyl orange) with varying concentrations of As(V) and Cd(II) (0–300 mg L^{-1}) were mixed. The final sample volume was 3 mL with 3 mg mL^{-1} Ft. After 24 h of stirring, the samples were centrifuged (20 000 rcf, 5 min) and the supernatant was collected and analyzed with ICP-OES. Samples without Ft were used as a reference.
The co-loading of oxo-anions and iron oxide was carried out by preparing an **aFt-P(10+)** crystal solution (2 mL, containing aFt 0.2 mg mL^{-1}) with 0.5 mM As(V) or Cd(II). The mixture was shaken in an open vessel to promote oxidation, and ten 100 µL aliquots of Fe(II) were added every 15 min to increase the final sample volume to 3 mL with 0.33 mM Fe(II). The samples were shaken overnight, centrifuged (20 000 rcf, 5 min), and the pellet washed twice with water. Finally, the pellet was dissolved into 150 mM NaCl, and centrifugation was repeated to remove any insoluble iron complexes. Finally, the samples were analyzed with ICP-OES. Samples without aFt were used as a reference.

Additional SAXS, DLS, and ζ-potential measurements

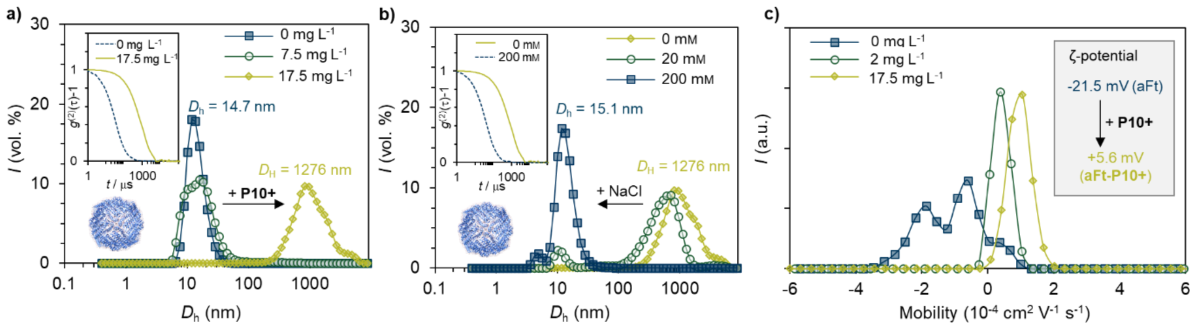

Figure S1: DLS size distribution profiles and ζ-potentials of aFt with **P10+**. a) DLS data for the volume-averaged size distribution of free aFt titrated with an increasing amount of **P10+** and b) the resulting complexes disassembled with NaCl. Insets: second-order autocorrelation functions of the corresponding measurements. c) Electrophoretic mobility and ζ-potential measured for **aFt–P10+** complexes

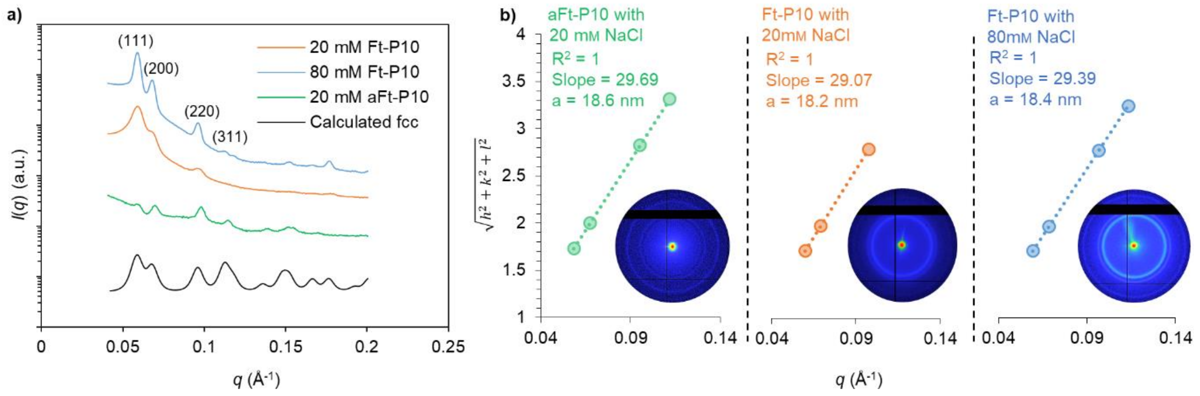

Figure S2: Additional SAXs calculations. (a) SAXS data compared to the theoretical fcc fitted model for selected complexes (data curves offset in y-direction for clarity). b) Miller indices of assigned reflections for the fcc structure versus measured q-vector positions for indexed peaks for **aFt-P10+** with 20 mM NaCl, **Ft-P10+** with 20 mM NaCl,

and **Ft-P10+** with 80 mM NaCl, which yielded unit cell dimensions of a = 18.6, 18.2, and 18.4 nm respectively (space group $Fm\bar{3}m$, number 225). Inset: 2D SAXS detector images.

Additional Optical microscopy and TEM images

Optical microscopy

The samples for optical microscopy were prepared using the hanging drop method (Figure S1). The CPF sample was prepared on top of a cover slip (18 x 18 mm, VWR) to hold the drop over the reservoir media, using the same concentration as for SAXS measurements. The reservoir media consisted of twice concentrated of buffer (*i.e.*, 40 mM Tris pH 7.5) and respective double concentration of NaCl (*e.g.*, for 10 mM NaCl in the sample, 20 mM NaCl concentration in the reservoir). 300 µL was poured into a screw cap. The coverslip on which the sample was prepared, was then attached to the reservoir cap using high vacuum glue in order to minimize evaporation of solvent. The sample droplet faced the closed side. The samples were incubated for two weeks at room temperature before imaging. Samples were imaged with Leica DM4500 P optical microscope equipped with Canon EOS 60D camera using transmission mode.

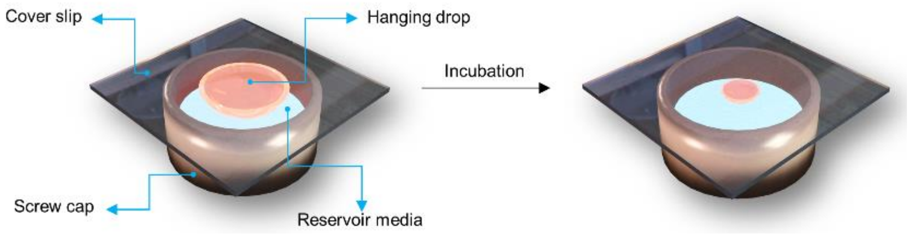

Figure S3: Schematic of hanging drop method.

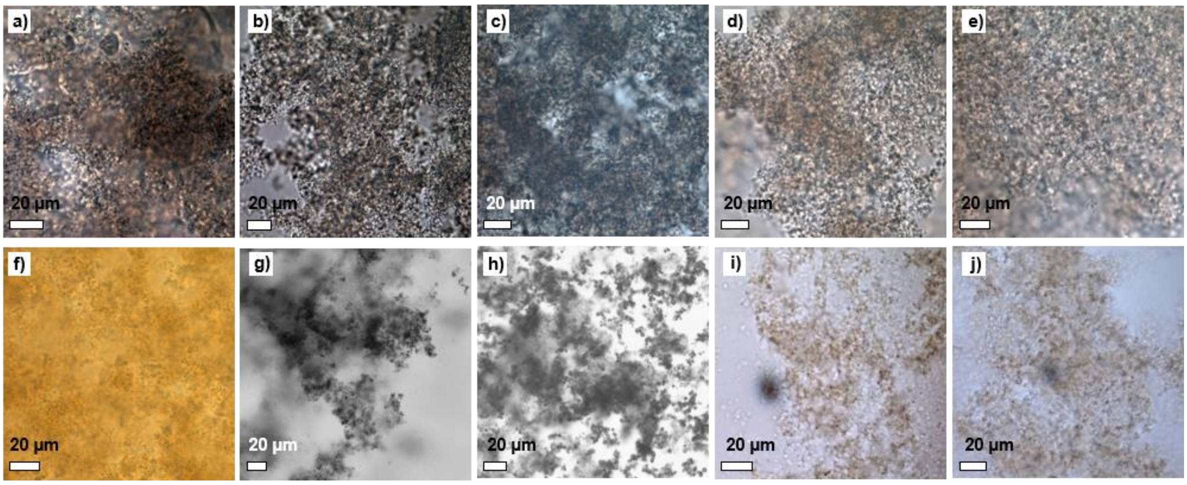

Figure S4: Low magnification optical microscopy images for **aFt-P10+** complexes at 0, 10, 20, 30, and 40 mM of NaCl (a–e, respectively); and **Ft-P10+** complexes at 0, 10, 30, 40, and 50 mM of NaCl (f–j, respectively).

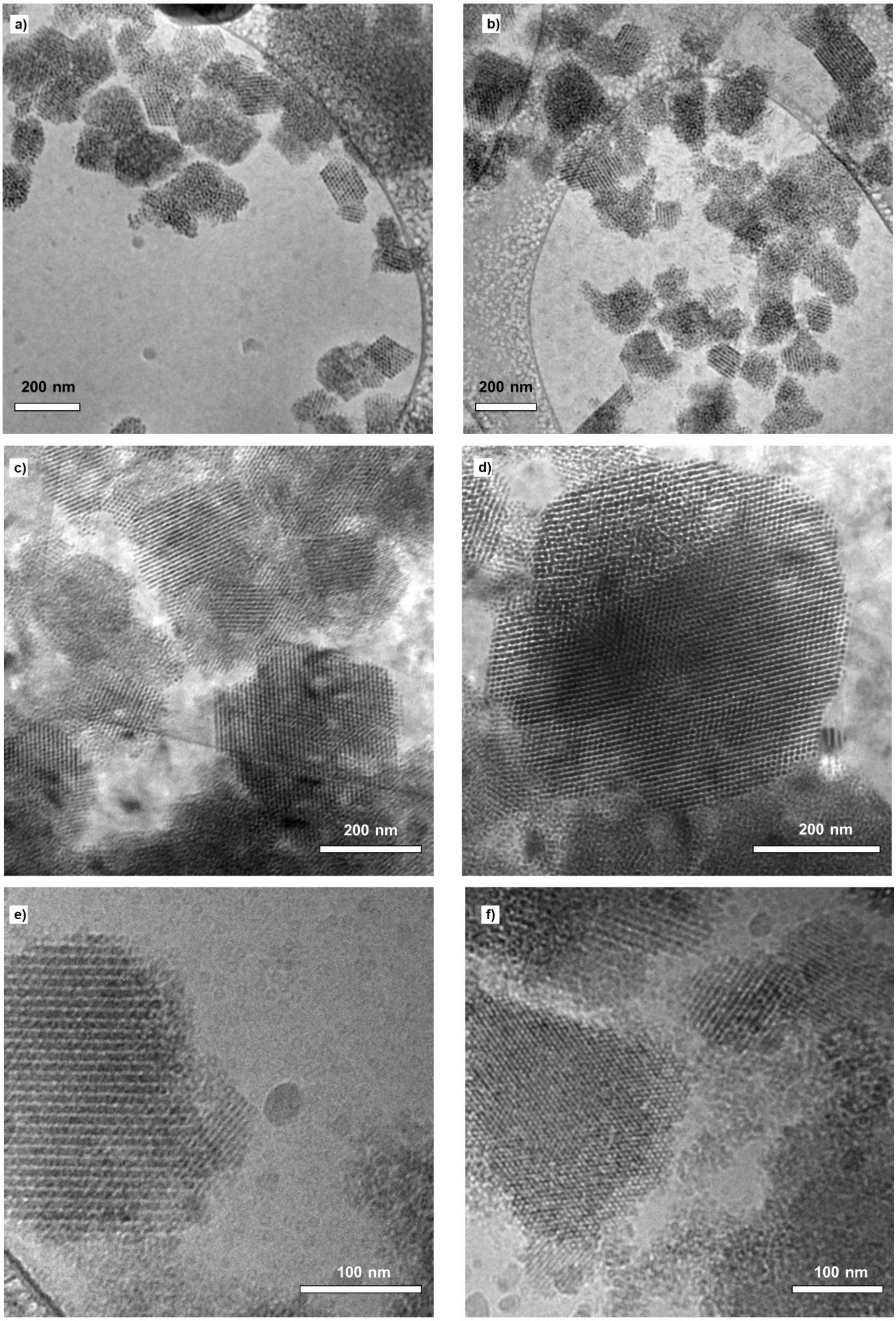

Figure S5: Low magnification and additional cryo-TEM micrographs for **Ft-P10+** with (a,b) 20 mM NaCl and (c,d) 80 mM NaCl and **aFt-P10+** (e,f) with 20 mM NaCl.

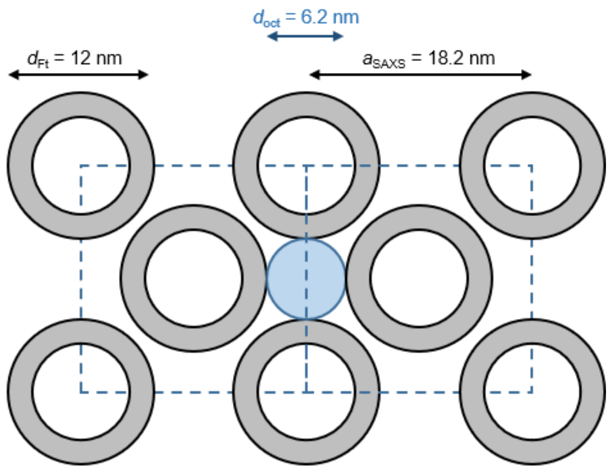

Figure S6: Pore size calculation through (100) plane for **Ft-P10+** with 20 mM NaCl using SAXS data. Grey rings represent Ft capsid, blue circle the octahedral void (d_{oct}). Iron core of Ft is not represented for clarity purposes.

Guest binding studied by NMR Spectroscopy

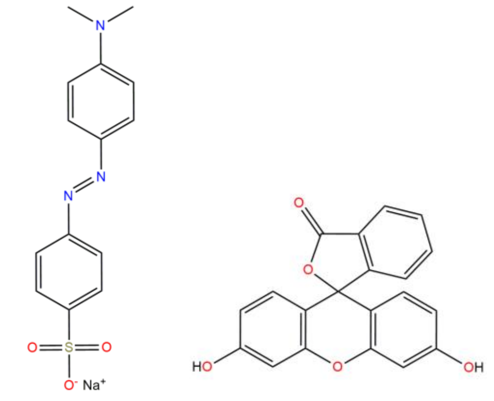

Figure S7: Guest chemical structures. Dyes methyl orange (MO) and fluorescein (FL).

Guest binding properties of the **P10+** receptor towards three organic dyes, methyl orange (MO) and fluorescein (FL) in solution was carried out through a series of NMR experiments. For sample preparation, 5 mM stock solutions of the **P10+** and the dyes were prepared. Due to the limited solubility of FL in D_2O, all the experiments were done in D_2O/[D_6]DMSO 10 % (v/v). Stock solution (0.5 mM) of **P10+** was prepared in pure D_2O. Stock solutions (4.5 mM) of the dyes were prepared in pure [D_6]DMSO. For the pure **P10+**, 450 µL of the stock solution was measured to an NMR tube and diluted with 50 µL of pure [D_6]DMSO to give a 0.45 mM sample concentration. For the pure dyes, 50 µL of the stock solution was measured to an NMR tube and diluted with 450 µL of pure D_2O to give a 0.45 mM sample concentration. For 1:1 host-guest mixtures, 450 µL of the **P10+** and 50 µL of each dye were measured to give a 0.45 mM concentration of both **P10+** and the dye.

a)

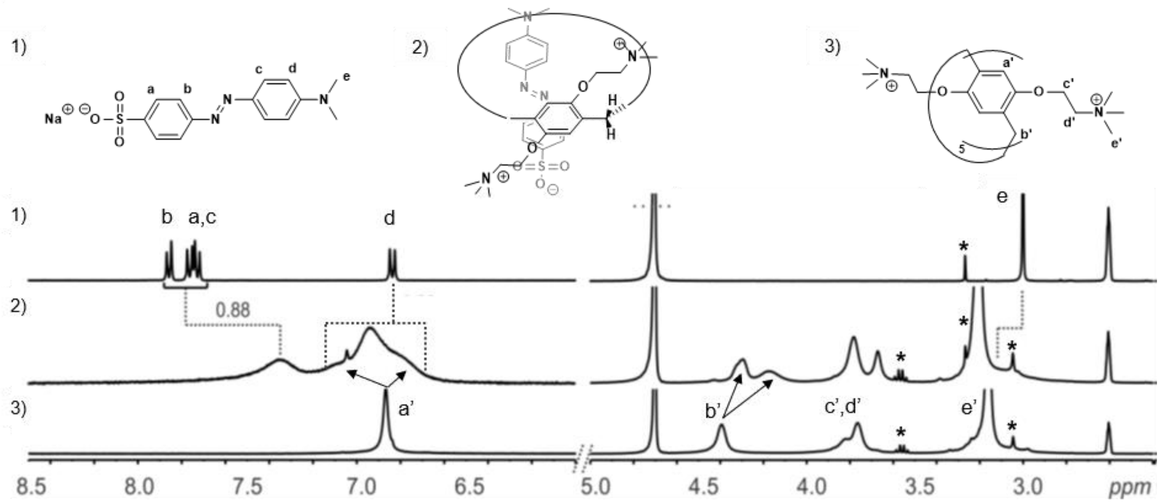

b)

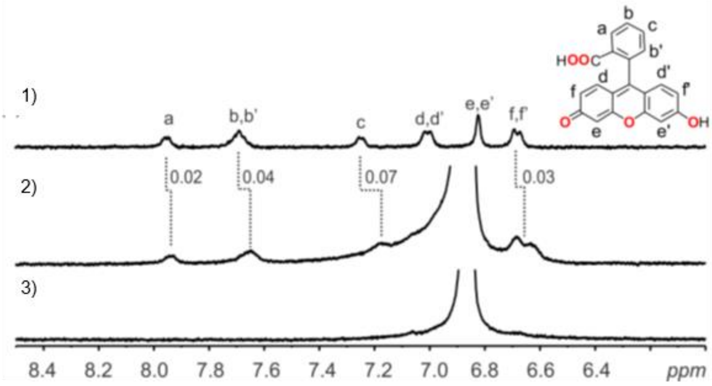

Figure S8: a) ^{1}H NMR spectra (D_2O/[D_6]DMSO 10 % v/v, 298 K) of: 1) MO, 2) equimolar mixture of **P10+** and MO, 3) **P10+**. The shift changes are presented in ppm, in dashed lines for the MO and arrows for **P10+**. * Trace impurities. b) ^{1}H NMR spectra (D_2O/[D_6]DMSO 10% v/v, 298 K) of: 1) P10+, 2) equimolar mixture of **P10+** and FL, 3) FL. The shift changes are presented in ppm.

Additional UV-Vis data

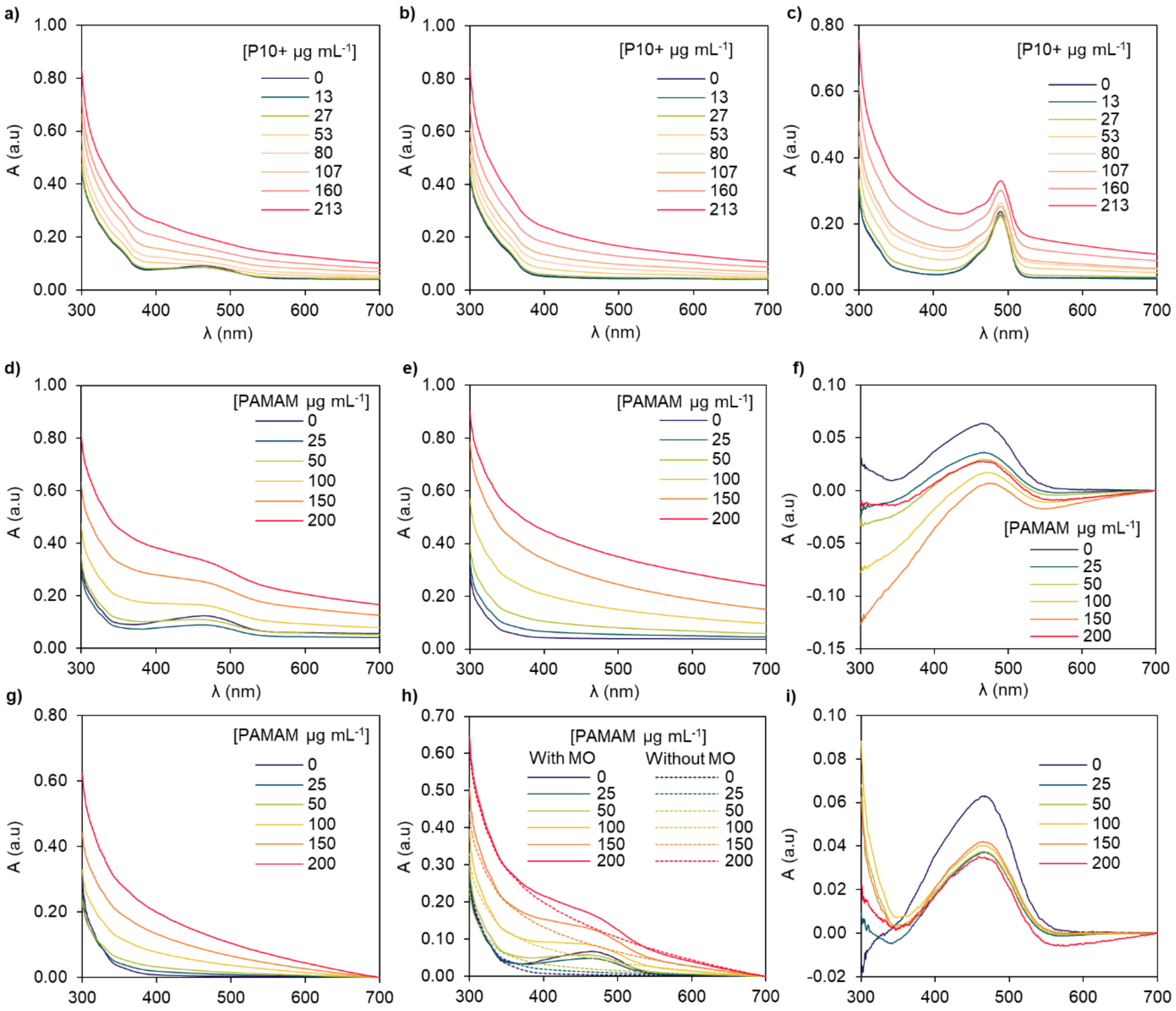

Figure S9: Host–guest binding studies using UV-Vis spectroscopy. a) Raw data (background not subtracted) for the titration of MO (2.5 μg mL^{-1}) with **aFt-P10+** crystals (C_{P10+} 0–213 μg mL^{-1}). b) Background titration of aFt with **P10+** (C_{P10+} 0–213 μg mL^{-1}) c) Raw data (background not subtracted) for the titration of FL (2.5 μg mL^{-1}) with **aFt-P10+** crystals (C_{P10+} 0–213 μg mL^{-1}) d) Raw data (background not subtracted) for the titration of MO (2.5 μg mL^{-1}) with **aFt-PAMAM** crystals (C_{PAMAM} 0–200 μg mL^{-1}). e) Background titration of aFt with PAMAM (C_{PAMAM} 0–200 μg mL^{-1}) f) Background substracted data for **aFt-PAMAM** (C_{PAMAM} 0–200 μg mL^{-1}) crystals with MO g) Multiplying the scattering value with a random factor (given below) to align the MO titration value of **aFt-PAMAM** crystals. h) Comparision of the scattering (dotted lines) with the MO titration (solid lines) of **aFt-PAMAM** crystals. i) substrating the aligned scattering values from MO titration of **aFt-PAMAM** crystals.

Multipliers for **aFt-PAMAM** crystals scattering for concentration 0, 25, 50, 100, 150 and 200 μg mL^{-1} are 1.2, 0.9, 0.7, 0.7, 0.7 and 0.95 respectively.

Isotherm parameters

Fitting the measured Cd and As adsorption data with Langmuir and Freundlich models yielded the following parameters (Table S1.)

Langmuir model: $q_e = q_{max}\frac{K_L c_e}{1+K_L c_e}$, and Freundlich model: $q_e = k{c_e}^{\frac{1}{n}}$,

where q_e is the bound concentration of metal in equilibrium, q_{max} is the maximum binding capacity at the studied conditions, K_L is the coefficient related to the free energy adsorption, c_e is the equilibrium concentration of metal, k is an indication of the relative adsorption capacity of the CPF and (1/*n*) is adsorption intensity.

Table S1: Isotherm parameters

	Langmuir			Freundlich		
	q_{max}	K_L	r^2	1/n	K_L	r^2
**Cd**	24.5	0.072	0.963	0.26	5.96	0.982
**As**	17.8	0.03	0.996	0.35	2.44	0.955

r^2 = 1-(RSS/TSS), where RSS is the residual sum of squares and TSS is the total sum of squares

Supplementary references:

[1] V. Liljeström, J. Seitsonen, M. A. Kostiainen, *ACS Nano* **2015**, *9*, 11278–11285.
